# Intubation of a Patient with a Large Goiter: The Advantageous Role of Videolaryngoscopy

**DOI:** 10.1155/2019/1327482

**Published:** 2019-12-05

**Authors:** Eric Heinz, Theodore Quan, Hieu Nguyen, Raymond Pla

**Affiliations:** ^1^Department of Anesthesiology and Critical Care Medicine, George Washington University Hospital, George Washington University School of Medicine and Health Sciences, 2300 I St NW, Washington, DC 20037, USA; ^2^George Washington University School of Medicine and Health Sciences, 2300 I St NW, Washington, DC 20037, USA

## Abstract

Fiberoptic bronchoscopy has long been considered the gold standard for patients who present with a difficult airway. In the case presented, a patient has a large palpable goiter and requires intubation. After the unsuccessful attempt to intubate with the use of fiberoptic bronchoscopy, the decision to switch to videolaryngoscopy afforded a positive result. We present this case to suggest that the utilization of videolaryngoscopy may be an alternative option for intubation when other methods have failed. It is imperative for anesthesiologists to understand the benefits that this modality may provide.

## 1. Introduction

Prior to the implementation of iodine supplementation in the 1920s, the rates of endemic goiters in the United States, especially in the Great Lakes, Appalachians, and the Northwest regions, were fairly high. Upon implementation of iodine, these numbers dropped drastically, from a reported rate of 26–70% to approximately 1–3% [1, 2]. Although the numbers are significantly smaller than they were a century ago, the presence of large goiters presents difficulties to the anesthesiologist in terms of airway management. According to the American Society of Anesthesiologists, a difficult airway is a clinical situation in which an anesthesiologist experiences difficulty with facemask ventilation of the upper airway or difficulty with tracheal intubation [3]. Cook et al. queried international experts in anesthetic airway management about their opinions regarding the best plan for a patient presenting with a thyroid mass which compressed her trachea. They found that the experts' opinions were strikingly different, indicating that there is no consensus on how to manage challenging airway cases [4].

In patients with clinically palpable goiters, the incidence of difficult intubation, which is defined as the inability to adequately expose the glottis with direct laryngoscopy, is approximately 13% [5]. Furthermore, if these goiters are complicated by additional airway deformity, the likelihood of difficult intubation increases [6]. These factors causes uneasiness among anesthesiologists with regards to airway management, ultimately leading to detailed evaluation and cautious management which slows case turnover [7]. In the current literature, fiberoptic bronchoscopy is touted as a tool that is able to circumvent a majority of the difficulties encountered in patients who have clinically palpable goiters. However, the case presented demonstrates the successful intubation of a patient with a longstanding clinically palpable goiter using videolaryngoscopy.

## 2. Case Report

**Figure 1:**
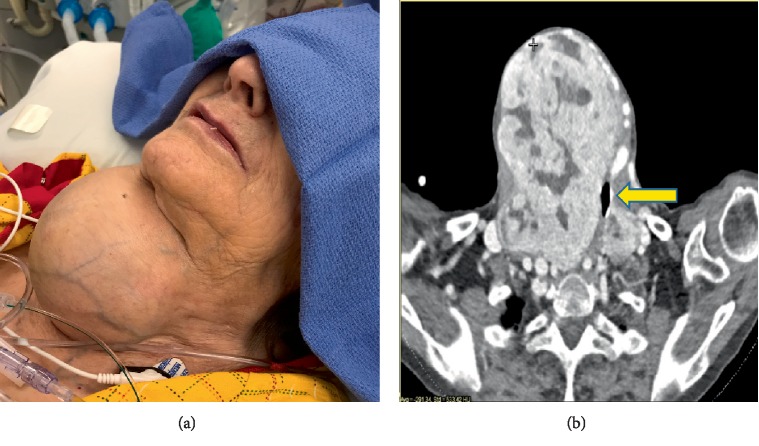
Image demonstrating the presence of the large clinically palpable goiter (a). CT scan showing trachea at its narrowest diameter (yellow arrow- (b)).

An 81 year-old woman (48 kg) presented with a right femoral neck fracture after a fall from ground level. She did not sustain any loss of consciousness or head trauma. Her past medical history was significant for dementia for which she required at-home care, and longstanding goiter which she did not recall having in childhood (Figure 1). No prior attempts at management of her goiter were documented. On physical exam, she was afebrile, normotensive, nontachycardic, with >95% oxygen saturation on room air. At baseline, she was alert and oriented only to self. She had no issues with phonation, but she did endorse shortness of breath while lying supine. No audible stridor was noted. The goiter was present over the entire anterior portion of the neck to below the sternal notch (8.8 × 12.0 × 10.1 cm). The goiter was firm, generally uniform, and immobile. On computed tomography (CT) scan, the goiter caused leftward tracheal deviation with a minimal tracheal diameter of 6 mm. Her Mallampati score was unable to be assessed given her dementia.

Prior to the procedure, a CT scan was obtained to elucidate tracheal diameter (6 mm at the narrowest point). Consent was obtained from patient's medical power of attorney after risks, benefits, and alternatives to anesthesia were obtained. Prior to proceeding to the operating room, the patient's vocal cords were sprayed with lidocaine 4% nebulizer, and the patient's nostrils were prepped bilaterally with oxymetazoline 0.05%. Dexmedetomidine drip was initiated for planned awake nasal fiberoptic intubation. Fiberoptic scope was passed through the nares, but the vocal cords were unable to be visualized due to the tongue, as well as debris from nasal passages. Even after readjusting the position and pulling the tongue anteriorly, there was still an inadequate view of the vocal cords. Jaw thrust maneuver yielded an improved view, however, a view of the vocal cords was unable to be maintained when attempting to pass the fiberoptic scope. Rightward pressure was then applied to the goiter in an attempt to bring the larynx into view without success. During this period, the patient became tachycardic necessitating an esmolol bolus.

Given the difficulty with the fiberoptic bronchoscopy, the decision was made to convert to videolaryngoscopy. While the patient remained sedated with a topically anesthetized airway, videolaryngoscopy revealed grade 3 Cormack-Lehane view. Eschmann stylet was advanced posterior to the epiglottis and tracheal grooves. Patient was given a bolus of 100 mg propofol to induce apnea. Armored 6.5 endotracheal tube was advanced over the Eschmann stylet and placement was confirmed with bilateral breath sounds as well as return of end tidal carbon dioxide on the ventilator. Anesthesia was maintained with sevoflurane. Over the course of the procedure, 1 mg of hydromorphone was administered for pain control. The patient required very small doses of pressors throughout the case and therefore she remained hemodynamically stable. The femoral neck fracture was repaired uneventfully. The patient remained intubated at the conclusion of the procedure due to concerns for airway edema given extensive airway instrumentation and possible tracheomalacia from her chronic goiter. She was kept sedated on propofol. On postoperative day 1, leak test was conducted which showed leak around the cuff. Dexamethasone was continued for airway edema. She was extubated successfully to BiPAP on postoperative day 3.

## 3. Discussion

The case presented describes a patient who had a femoral neck fracture after a fall. The need to intubate was necessary for the patient, despite the presence of a goiter. Multiple studies have examined the relationship between the presence of a goiter and difficulty with intubation as assessed by the Intubation Difficulty Scale (IDS). In one such study, there was an 11.1% rate of difficult intubation in patients with goiters undergoing thyroid surgery [5]. However, in another study, the rate was calculated to be 5.3% [8]. Ultimately, the presence of a goiter has not been found to increase the IDS score. Nevertheless, the presence of malignant goiters has correlations to difficult intubations. Other risk factors include tracheal stenosis or deviation, and reduced mouth opening [9, 10]. These factors are in addition to the commonly known predictors of difficult intubation: Cormack-Lehane 3 or 4, increased neck circumference, and age >55 [11].

When it comes to airway management for patients with clinically apparent goiters, there are multiple options for the anesthesiologist. Methods such as awake direct laryngoscopy and awake rigid bronchoscopy have fallen by the wayside, whereas methods using awake fiberoptic intubation or awake tracheostomy are still in use [12]. Other methods such as cardiopulmonary bypass or extracorpeal membrane oxygenation via femoral vessel cannulation are available but only in specialized hospitals that manage this type of patient. Many papers champion the use of fiberoptic intubation for potentially difficult intubations, including the patient population that was addressed in this report. However, a recent meta-analysis from Alhomary et al. aggregated 8 studies with 429 patients. In this study, failure rate and first attempt success rate between the 2 modalities, videolaryngoscopy and fiberoptic bronchoscopy, were not statistically significant. It was seen that the use of videolaryngoscopy yielded a faster time to tracheal intubation than fiberoptic bronchoscopy [13, 14]. In another study by Grgurich et al., there was no significant difference in time to tracheal intubation and first attempt success between videolaryngoscopy and awake fiberoptic intubation [15]. Other papers have alluded to the use of a combination of the above-mentioned methods, usually after failure to intubate by conventional means. One such case report details the successful use of a McGrath MAC videolaryngoscope for improved airway visualization with fiberoptic bronchoscopy as a stylet to pass an endotracheal tube. This was performed after the failure of direct laryngoscopy and videolaryngoscopy, as well as awake fiberoptic bronchoscopy [16].

In the emergency departments and intensive care units, videolaryngoscopy is becoming routinely used as the first attempt during intubation. Consequently, it may be beneficial for anesthesiologists to begin to also adopt this practice [17]. Despite the benefits of the utilization of videolaryngoscopy, there are several disadvantages with the use of this tool. It has been demonstrated that intubation using videolaryngoscopy does not allow for continuous ventilation to occur unlike the other modalities [18, 19].

This case illustrated the unsuccessful use of fiberoptic bronchoscopy for intubation in the patient with a large goiter. The decision to switch to videolaryngoscopy yielded a fruitful outcome. Although videolaryngoscopy is an excellent tool, there is no clear evidence that it is superior to the other various tools in every clinical situation, and therefore, the practitioner should use this modality at his discretion. The anesthesiologist must recognize the vast array of tools at his disposal and ultimately utilize what is best for the patient's situation. More studies need to be completed to compare the various methods for the management of patients with a difficult airway.
